# Laminar flow drag reduction on soft porous media

**DOI:** 10.1038/s41598-017-17141-3

**Published:** 2017-12-08

**Authors:** Parisa Mirbod, Zhenxing Wu, Goodarz Ahmadi

**Affiliations:** Department of Mechanical and Aeronautical Engineering, Clarkson University, Potsdam, New York, United States

## Abstract

While researches have focused on drag reduction of various coated surfaces such as superhydrophobic structures and polymer brushes, the insights tso understand the fundamental physics of the laminar skin friction coefficient and the related drag reduction due to the formation of finite velocity at porous surfaces is still relatively unknown. Herein, we quantitatively investigated the flow over a porous medium by developing a framework to model flow of a Newtonian fluid in a channel where the lower surface was replaced by various porous media. We showed that the flow drag reduction induced by the presence of the porous media depends on the values of the permeability parameter α = L/(MK)^1/2^ and the height ratio δ = H/L, where L is the half thickness of the free flow region, H is the thickness and K is the permeability of the fiber layer, and M is the ratio of the fluid effective dynamic viscosity μ_e_ in porous media to its dynamic viscosity μ. We also examined the velocity and shear stress profiles for flow over the permeable layer for the limiting cases of α → 0 and α → ∞. The model predictions were compared with the experimental data for specific porous media and good agreement was found.

## Introduction

**Figure 1:**
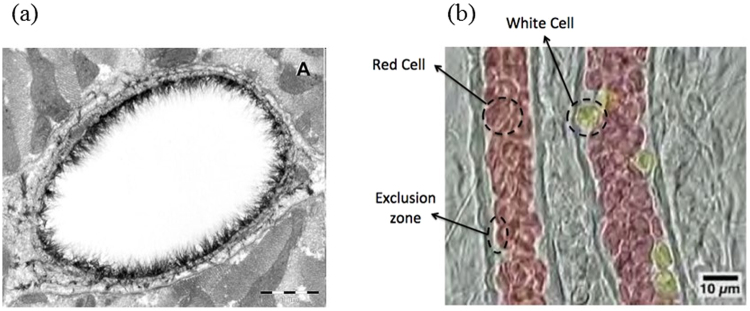
(**a**) Endothelial glycocalyx of a rat left ventricular myocardial capillary stained with Alcian blue 8GX and visualized using electron microscopy. Bar represents 1 μm. (Reproduced from^50^); (**b**) Motion of red and white blood cells in an arteriole^51^.

The possibility of using soft, porous, highly compressible materials as lubricating layers is a new concept that has grown out of several fundamental biological questions related to the role of the fiber matrix layers that surround cells in an *in vivo* fluid environment^1^. In fact, experimental studies of blood flow in microvessels revealed differences from analytical predictions based on *in vitro* observations^2^. It was found that the main reason for these differences is the presence of a relatively thick (~1 μm) layer of macromolecules bound to the endothelial cells lining microvessel walls^3^. This layer, which is located on the luminal surface of vascular cells, is called the endothelial glycocalyx layer (EGL) (Fig. 1a). While the existence of the surface glycocalyx has been known for several decades^4^, only in the past few years has it been appreciated as an important factor in vascular physiology and pathology^5^. Computer-enhanced images showed that the glycocalyx is a 3D fibrous mesh network. Using a freeze-fracture method, it was also shown that the fibers formed a hexagonal array with an inter-cluster spacing of typically 100 nm in frog lung capillaries^6^.

In addition to preventing adhesive molecular interaction between proteins in the red cell membrane and their ligands in the plasmalemma membrane of the endothelial cell, the EGL provides a red blood cell “exclusion zone” or “gap” between the flowing red blood cells and the endothelial membrane as shown in Fig. 1b. It was also shown that the EGL reduces the volume fraction of the red blood cells and affects interactions of blood cells with vessel walls^7^.

Feng and Weinbaum^8^ further found the so-called “pop out” phenomenon in which at velocities <20 μm/s the red cells entered the EGL and, when motion was arrested, the red cells crushed the glycocalyx and filled nearly the entire lumen of the capillary. At velocities >20 μm/s, the red cells appeared to glide above the EGL, and there was a narrow intervening fluid gap. To describe the latter motion, a lubrication theory for soft porous highly compressible materials with periodic fiber arrays was developed^8^. Later a lubrication theory for randomly oriented soft porous media was also established^9^. Using the proposed lubrication theory, it was shown that the corresponding generated lift forces could be four orders of magnitude greater than those predicted by classical lubrication theory^9–11^.

The concept of the almost frictionless movement of red blood cells through tiny capillaries and the impact of the EGL on the red blood cell motion inspired us to examine a totally different scenario: analysis of the laminar skin friction coefficient and drag reduction in micorchannels where the bottom solid surface is replaced with a layer of porous material with permeability K and porosity ε. While the topic of drag reduction has been the focus of recent research on surfaces coated with a broad array of structures such as superhydrophobic structures and polymer brushes, the insights to understand the fundamental physics of the laminar skin friction coefficient and the related drag reduction due to the formation of a slip velocity at the surface of a porous media boundary in the pressure-driven flow is still not fully understood. Herein, we focus on answering the questions including: 1) What is the relation between the characteristics of the porous media (i.e., permeability, and porosity) and the geometry of the channel with the resulting drag on the porous walls? 2) Can replacing an impermeable wall with a permeable surface be used as a new passive technique for reducing friction in a pressure-driven duct flow? 3) How the characteristics of porous media and channel geometry can be correlated to the drag force on the wall?

Coupled flows through and over porous layers have been covered in several papers. A great number of publications^12–16^ provide reviews of related research developments in the literature. Researchers have investigated flow over sediment beds^17^, coral reefs and submerged vegetation canopies^18^, crop canopies and forests^19^, endothelial glycocalyx of blood vessels^20^, flow over carbon nanotubes (CNTs)^21^, and polymer brushes^22,23^, and the finite-Reynolds flow in a channel bounded by one or two porous media modeled by cubic sphere arrays^24^. The drag reduction due to the motion of a viscous fluid, in laminar and turbulent flows, over CNTs, over patterned surfaces, and over various other structured surfaces has been examined experimentally and theoretically^25–46^. However, to the best of our knowledge, no one has ever attempted to characterize the laminar drag reduction in a channel in the presence of porous walls. In this study, we present analytical description of the laminar skin friction coefficient and the corresponding drag reduction of the flow at the fluid-porous interface inside a channel with the lower surface replaced with various porous layers with Darcy permeability K, and porosity ε. The conditions for which the porous materials can be used as a new passive technique for reducing drag in laminar flow regimes were also described.

## Mathematical formulation

**Figure 2:**
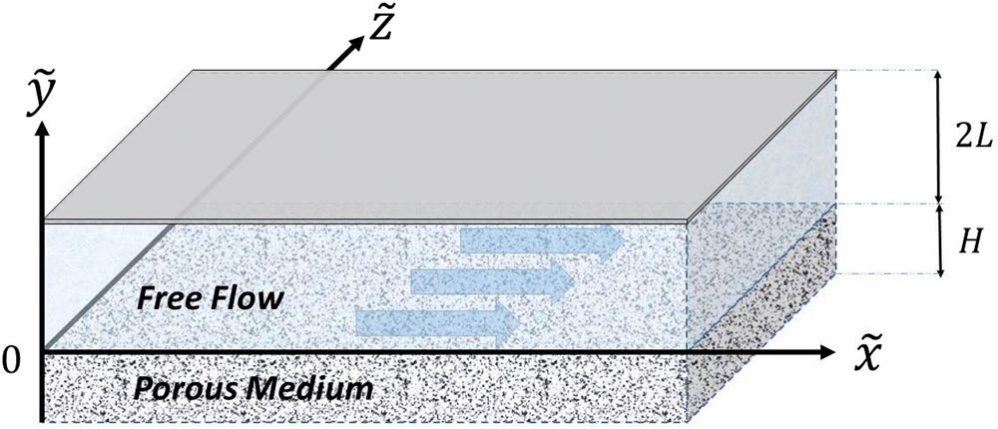
Schematic diagram of a fully developed flow in a rectangular channel over a permeable surface with permeability K and porosity ε. Arrows indicate the flow direction.

In this section, we consider a fully developed, incompressible flow driven by a constant pressure gradient $d\tilde{{\rm{p}}}/d\tilde{{\rm{x}}}$ within a two-dimensional (2D) channel where H is the thickness of a porous layer with permeability K and porosity ε and 2 L is the height of the free flow. The two regions of a core flow and a flow through the porous layer are sketched in Fig. 2. The effective medium approach (Brinkman equation) is used for flow through the porous medium and the flow in the core region is described by the Navier-Stokes equation for unidirectional axial flow in a rectangular channel. For this fully-developed flow, the inertial terms vanish. Thus, the simplified equations in the laminar regime can be expressed as

1 $${\mu }_{e}\frac{{d}^{2}\tilde{u}}{d{\tilde{y}}^{2}}-\frac{\mu }{K}\tilde{u}-\frac{d\tilde{p}}{d\tilde{x}}=0,\tilde{y}\in [-H,0]\,$$

2 $$\mu \frac{{d}^{2}\tilde{u}}{d{\tilde{y}}^{2}}-\frac{d\tilde{p}}{d\tilde{x}}=0,\tilde{y}\in [0,2L]\,$$

where $\tilde{u}$represents the horizontal component of the velocity throughout the channel, *μ* is the dynamic viscosity of the fluid, and *μ*_*e*_ is the effective viscosity of fluid in the porous layer. The effective viscosity in porous media is widely discussed in previous studies^47^. The conclusion of these earlier studies is that for high porosity material, *μ*_*e*_ → *μ*. The boundary conditions on the two walls of the channel, namely at $\tilde{y}=-H$ and $\tilde{y}=2L$ need to be specified. We then require that the no-slip boundary condition to be satisfied, that is $\tilde{u}(-H)=0$ and $\tilde{u}(2L)=0$. In addition, the boundary conditions at the fluid-porous layer interface must be prescribed. Herein, following the researchers^21,48^ we require that the velocity and fluid shear stress to be continuous at the fluid-porous interface (i.e., at $\tilde{y}=0$). The continuity of velocity and shear stress at the fluid-porous interface implies that $\tilde{\,u}({0}^{-})=\tilde{u}({0}^{+})=\tilde{U},{\mu }_{e}{(\frac{d\tilde{u}}{d\tilde{y}})}_{\tilde{y}={0}^{-}}=\mu {(\frac{d\tilde{u}}{d\tilde{y}})}_{\tilde{y}={0}^{+}}$ where, $\tilde{U}$is the velocity at the fluid-porous interface.

In addition, the half of the height of the free fluid, L, the velocity $q=-(\frac{{L}^{2}}{\mu })\frac{d\tilde{p}}{d\tilde{x}}$ and the fluid viscosity, *μ* are chosen as the repeating variables to analyze a wide range of parameter changes in the system as well as to resolve the infinite solutions. Thus, the solutions for the dimensionless velocity profile,$\,u=\tilde{u}/q$, over and through the soft porous media can be given by

3 $$u(y)=\frac{1}{M{\alpha }^{2}}+{C}_{1}{e}^{\alpha y}+{C}_{2}{e}^{-\alpha y},y\in [-\delta ,0]$$

4 $$u(y)=-\frac{{y}^{2}}{2}+(1-\frac{U}{2})y+U,y\in [0,2]\,$$

5 $${C}_{1,2}=\pm \frac{1}{M{\alpha }^{2}}\frac{(M{\alpha }^{2}U-1){e}^{\pm \alpha \delta }+1}{{e}^{\alpha \delta }-{e}^{-\alpha \delta }}\,U=\frac{1-\,{\rm sech} \,\alpha \delta +\alpha \,\tanh \,\alpha \delta }{\beta M{\alpha }^{2}}\,$$

6 $$\beta =(1+\frac{\tanh \,\alpha \delta }{2M\alpha })$$

where

7 $$y=\frac{\tilde{y}}{L},M=\frac{{\mu }_{e}}{\mu },{\alpha }^{2}=\frac{{L}^{2}}{MK},\delta =\frac{H}{L},U=\tilde{U}/q$$

Moreover, the fluid shear stress in the free flow and inside the soft porous material can be described as,$\,\tilde{\tau }=\mu \frac{d\tilde{u}}{d\tilde{y}}$ when $\tilde{y}\in [0,2L]$ and $\tilde{\tau }={\mu }_{e}\frac{d\tilde{u}}{d\tilde{y}}$ for $\tilde{y}\in [-H,0]$ and in the dimensionless form they can be expressed as $\tau =L\tilde{\tau }/\mu q$. Note that the solid shear stress inside the porous media is not evaluated in the present study. Thus, the fluid shear stress in the pressure-driven channel flow with the solid wall replaced with soft porous media can be obtained as

8 $$\tau (y)=M(\frac{du}{dy})=M\alpha ({C}_{1}{e}^{\alpha y}-{C}_{2}{e}^{-\alpha y}),y\in [-\delta ,0]$$

9 $$\tau (y)=(\frac{du}{dy})=(-y+1-\frac{U}{2}),y\in [0,2]\,$$

Evaluating the fluid shear stress at the fluid-porous medium interface,$\,{\tilde{\tau }}_{w}=\mu {\frac{d\tilde{u}}{d\tilde{y}}|}_{\tilde{y}={0}^{+}}$, the skin friction coefficient is given as ${C}_{f}=2{\tilde{\tau }}_{w}/\rho {\tilde{u}}_{ave}^{2}$. Herein, Re is the Reynolds number in the core free flow region defined as $\,Re=2L{\tilde{u}}_{ave}/\nu$, where *v* is the kinematic viscosity of the fluid and ${\tilde{u}}_{ave}=q{u}_{ave}\,\,\,$ is the average velocity of the flow through the non-porous section where the dimensionless average velocity is given by ${u}_{ave}=\frac{{\int }_{0}^{2}u(y)dy}{2}$. Thus, the skin friction coefficient due to the presence of the soft porous wall can be defined as *C*_*f*_ = *A*/*Re*, where $A=\frac{8(1-U/2)}{{\int }_{0}^{2}u(y)dy}$ is a constant. This equation provides a closed-form expression for the skin friction coefficient, *C*_*f*_ when the properties of the soft porous media such as permeability, the channel geometry and the flow conditions are identified.

It should be noted that these results simply indicate that for δ = *H*/*L* = 0 we obtain the same results as in a channel with impermeable walls, where the dimensionless velocity profile is given by $u(y)=-{y}^{2}/2+y,$ $y\in [0,2]$. Thus, the well-known skin friction formula ${C}_{{f}_{s}}=\frac{12}{Re}$ for laminar flows in two-dimensional channel with smooth solid walls is obtained^49^. One can then calculate the laminar drag reduction for a channel with one impervious wall replaced with soft porous media as

10 $$DR \% =100(1-\frac{{C}_{f}}{{C}_{{f}_{s}}}) \% =100(1-\frac{A}{12}) \% .$$

### Special cases analysis

The asymptotic behavior of the flow is examined in this section. When α → ∞, the velocity and shear stress profiles, and the constant of friction coefficient reduce to

11 $$\mathop{{lim}}\limits_{\alpha \to \infty }u(y)=\{\begin{array}{ll}\frac{y(2-y)}{2} & y\in [0,2]\\ 0 & y\in [-\delta ,0]\end{array}\,$$

12 $$\mathop{\,{lim}}\limits_{\alpha \to \infty }\tau (y)=\{\begin{array}{ll}1-y & y\in [0,2]\\ \,0 & y\in [-\delta ,0]\end{array}\,$$

13 $$\mathop{{lim}}\limits_{\alpha \to \infty }A=12\,y\in [-\delta ,2]$$

These results show that when α → ∞, the velocity profile and skin friction coefficient asymptotically approach to the expressions for the pressure-driven flow in a channel with impermeable walls and dimensionless height of 2, and there will be no drag reduction.

In the limit when α → 0, which corresponds to an infinite value of K, the expressions for velocity and shear stress profiles, and the constant of friction coefficient simplify to

14 $$\mathop{{lim}}\limits_{\alpha \to 0}u(y)=\frac{(\delta +y)(2-y)}{2}\,y\in [-\delta ,2]$$

15 $$\mathop{{lim}}\limits_{\alpha \to 0}\tau (y)=1-y-\delta /2\,y\in [-\delta ,2]$$

16 $$\mathop{{lim}}\limits_{\alpha \to 0}A=\frac{12(2-\delta )}{3\delta +2}\,y\in [-\delta ,2]$$

Eqs. (14–16) reveal that for the case when α → 0, there are significant drag reduction depending on the value of *δ.* For *δ* = 2 the wall shear stress at the fluid-porous interface at y = 0 becomes zero and the maximum drag reduction of 100% will be achieved.

## Results and Discussion

**Figure 3:**
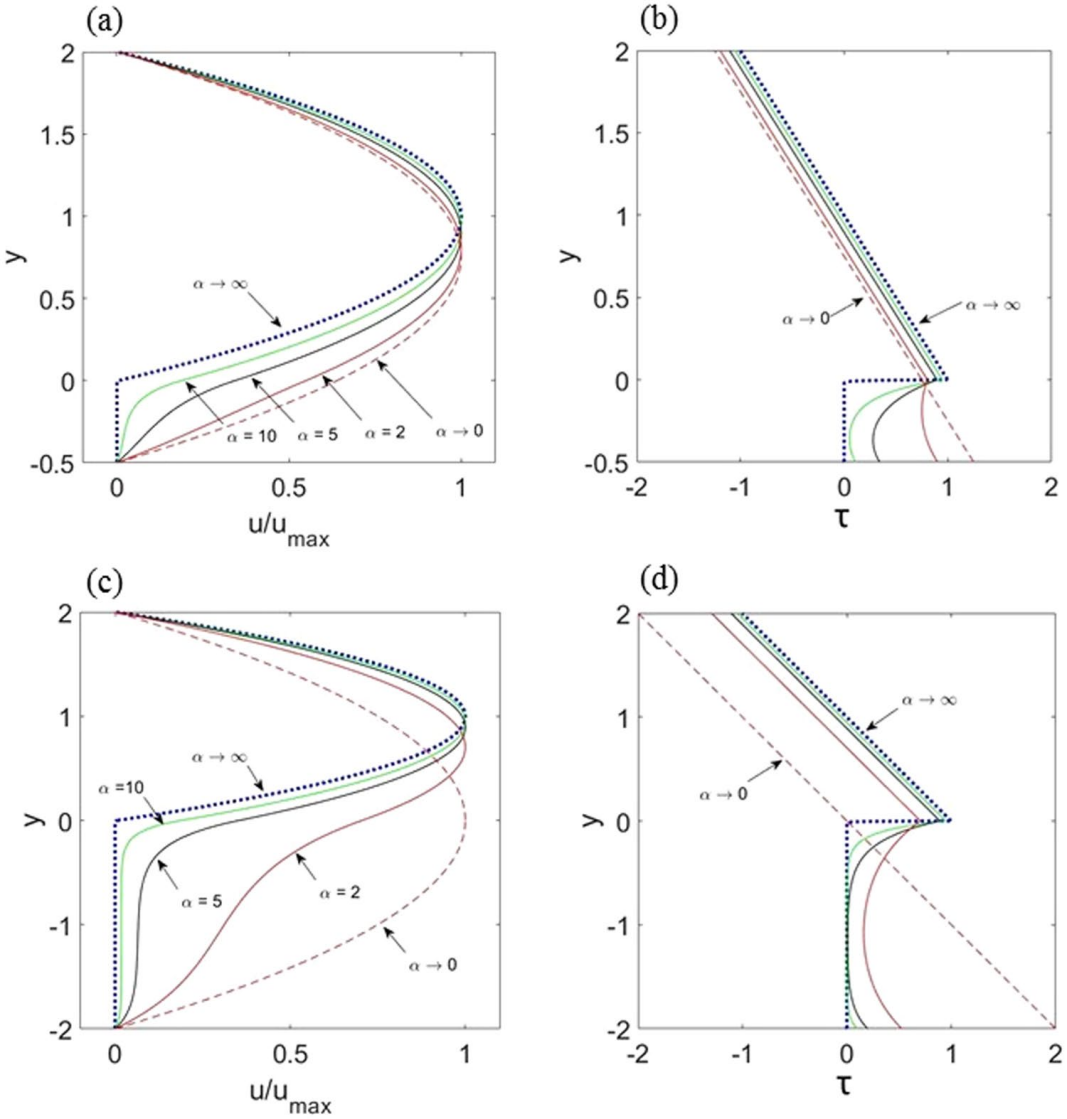
Variations of normalized velocity and dimensionless fluid shear stress profiles in the core region and inside the porous media for α = 2,5,10 and the asymptotic solutions in the limit α → ∞ (dotted line) and *α* → 0 (dashed line). Here (**a**,**b**) δ = H/L = 0.5 and (**c**,**d**) δ = H/L = 2. For all cases M = 1.

The fluid velocity and shear stress profiles across the channel including inside the porous layer as predicted by equations (3–9), for two fiber layer thicknesses, are shown in Fig. 3(a–d). In the large α = L/(MK)^1/2^ limit (α ≥ 10), the core velocity profile is parabolic except near the interface, where the velocity drops to very small values. In addition, for large values of α, when the permeability of the porous material becomes low, the velocity profile inside the porous layer shows the characteristic of plug flow, except near the free fluid-porous medium interface (Fig. 3a,c). Also, for large α values the fluid shear stress in the porous layer decays to zero. This is because as the permeability decreases, the bulk of shear stress is sustained by the fibers in the porous layer, which results in very small velocity and fluid shear stress (Fig. 3b,d). For comparison, the asymptotic solutions of equations (11), (12), (14), and (15) in the limits of α → ∞ and α → 0 are shown, respectively, by dotted lines and dashed lines in Fig. 3. These results clearly show that as α → ∞ the velocity and shear stress profiles asymptotically approach to the outcomes of the pressure-driven flow in a channel with solid smooth walls and a height of 2 L. Similarly, when α → 0, the velocity and shear stress profiles become those for a clear channel with the height of 2 L + H.

**Figure 4:**
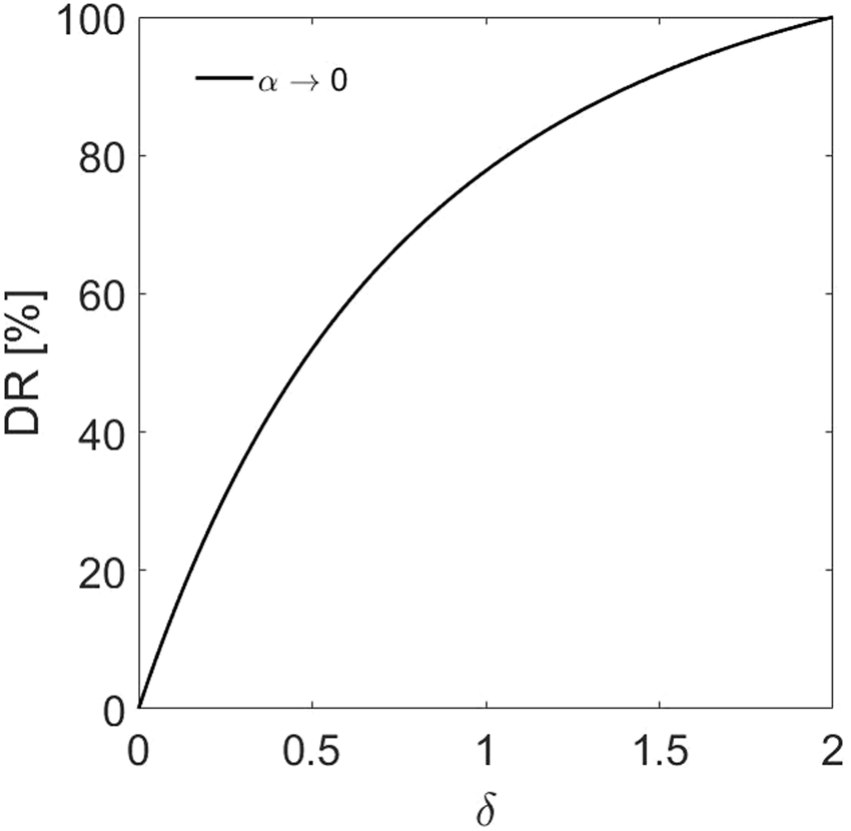
Drag reduction, DR% in a channel coated with soft permeable media as a function of δ for *α* → 0 and M = 1.

Figure 4 shows the variation of the drag reduction, DR%, versus δ when α → 0. This figure reveals that as δ increases, the drag reduction in the channel increases and at δ = 2, the maximum drag reduction of 100% is achieved. Thus, the implication of this figure is that the maximum drag reduction occurs in a system when the height of the free flow region is the same as the thickness of the porous layer (i.e., δ = 2). Also, in this case we have the highest permeable porous media as a boundary (i.e., K ≈ ∞). Accordingly, all subsequent calculations are presented for δ ranging from 0 to 2.

**Figure 5:**
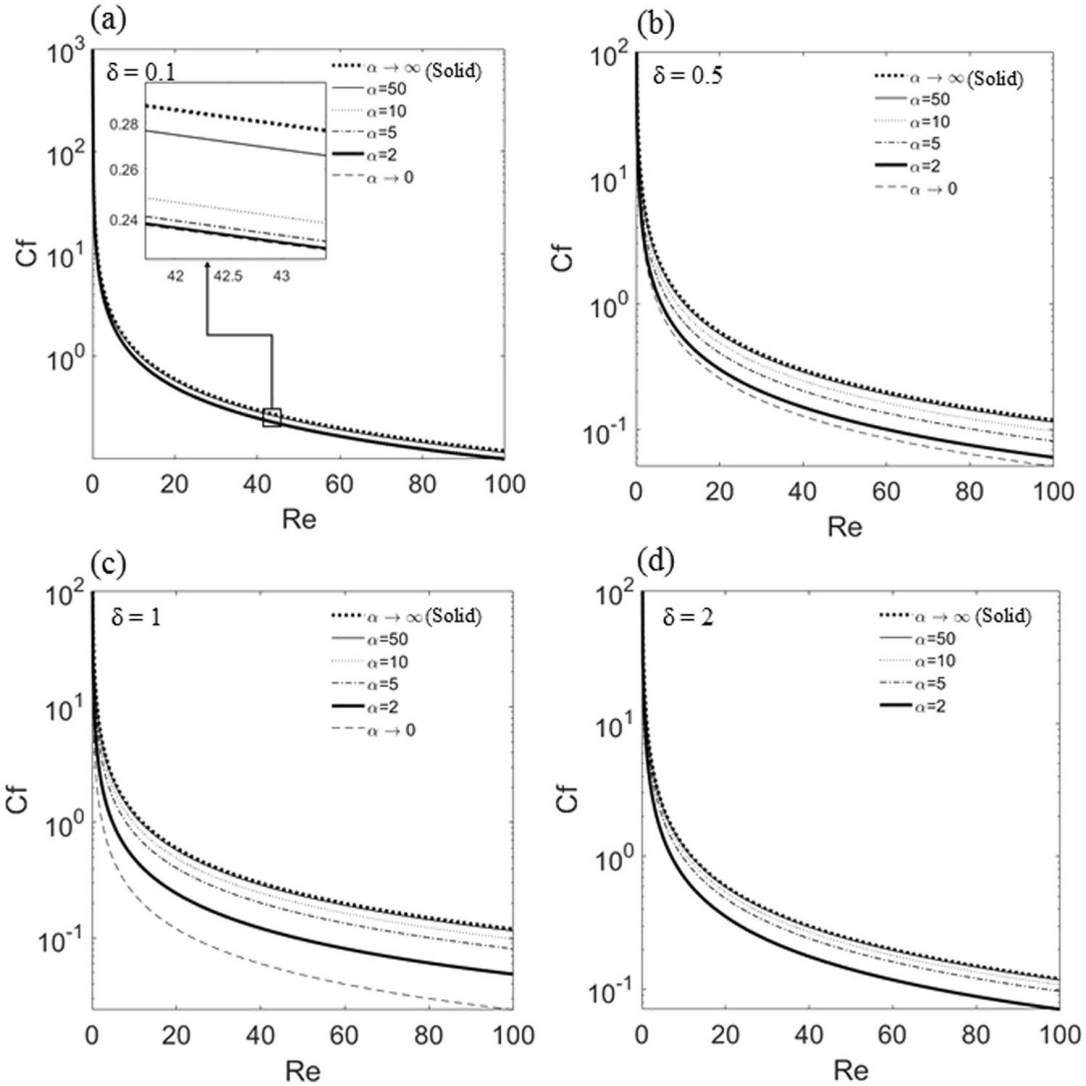
Variations of skin friction coefficient, C_f_, as a function of Re for different values of *α* and M = 1. (**a**) δ = 0.1; (**b**) δ = 0.5; (**c**) δ = 1; (**d**) δ = 2. Dotted and dashed lines show the asymptotic solutions of C_f_ in the limit α → ∞ and α → 0, respectively.

Figure 5(a–d) show the variation of laminar skin friction coefficient versus the Reynolds number for different values of α at δ = 0.1, 0.5, 1, and 2. For comparison, the prediction of the friction coefficient for the channel with solid impermeable walls, ${C}_{{f}_{s}}=\frac{12}{Re}\,\,$, is reproduced in these figures by dotted lines, which also coincide with the asymptotic solution of C_f_ in the limit when α → ∞. Similarly, the asymptotic solution of C_f_ in the limit when α → 0 is shown by dashed lines. As can be seen in Fig. 5(a–d), for all values of δ and the Reynolds number ranging from 0 to 100, the skin friction coefficient for various porous layers are found to lie well below that of the channel with solid walls (α → ∞). Figure 5(a–d) shows that for all values of the height ratio δ, and permeability parameter α, a finite slip velocity is generated at the fluid-porous interface, which helps the fluid motion through the channel and reduces the drag. It is also seen that the skin friction coefficient decreases as Re increases and/or α decreases. The sensitivity of the variation of C_f_ with α also varies depending on the thickness of the porous layer. It should be noted that as the height ratio, δ increases to a value of 2, in the limit when α → 0, C_f_ becomes zero and this result could not be shown in a log-plot in Fig. 5d.

**Figure 6:**
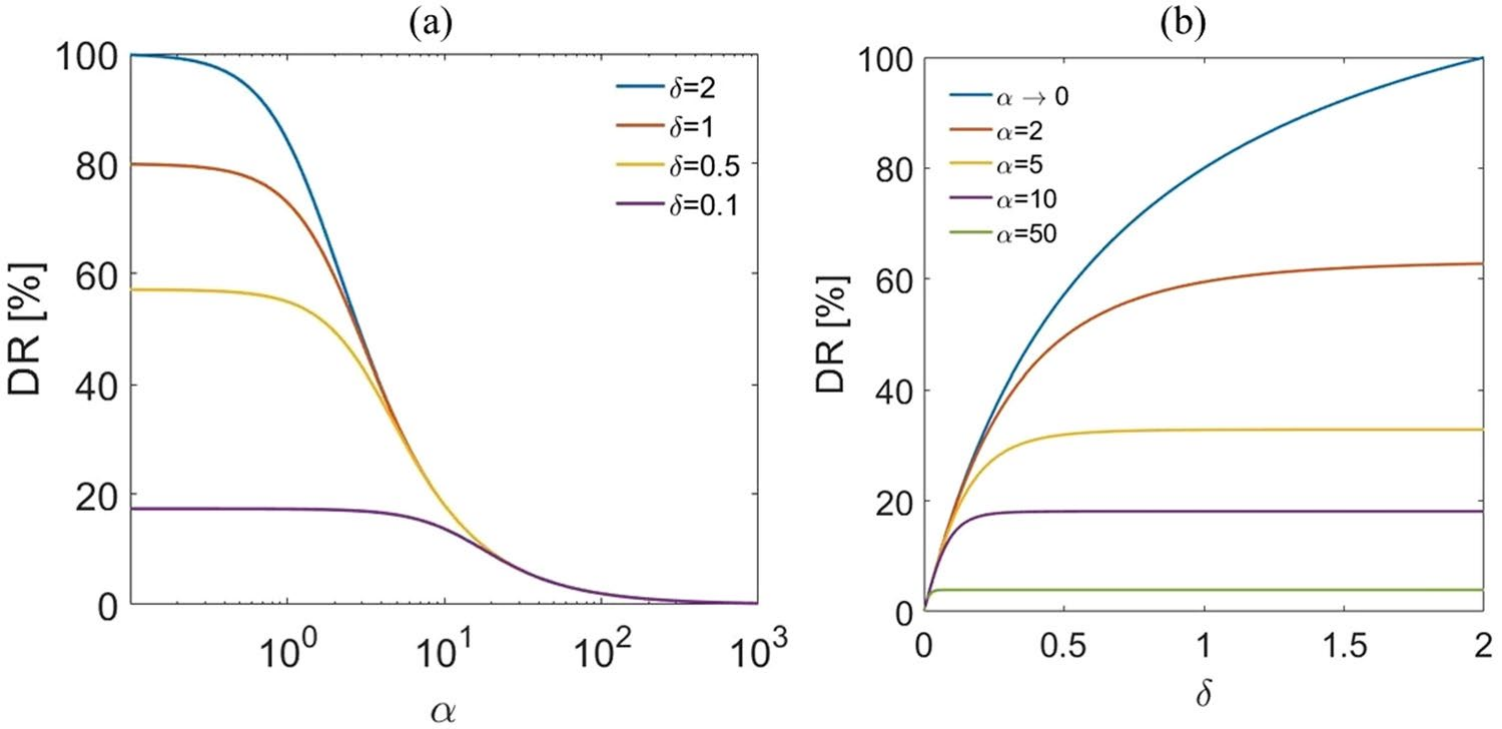
(**a**) Drag reduction as a function of *α* for different *δ* values. (**b**) Drag reduction as a function of *δ* for different *α* values. For all case M = 1.

For a more clear comparison, variations of drag reduction DR% versus *α* for different δ values are shown in Fig. 6a. This figure shows that as *α* increases the drag reduction decays to zero for all values of δ. Also, as δ increases the drag reduction increases. For small values of *α* corresponding to high permeability, even for small thicknesses of porous wall, there are meaningful drag reductions in the system. However, for large values of *α* (low values of permeability), the porous-wall surface would not provide much drag reduction in the channel even for large values of δ. As the height ratio increases to 2 where the height of the free-flow and the thickness of the fiber layer are the same, the maximum drag reduction in the system occurs.

The drag reduction, DR%, as a function of δ for various permeability parameters *α* are plotted in Fig. 6b. This figure shows that the amount of drag reduction in the channel with a porous wall depends strongly on the values of δ and α. When permeability parameter α decreases and/or the height ratio δ increases, an increase in the drag reduction is predicted. As α decreases further, the drag reduction approaches to the asymptotic solution for α → 0. Figure 6b also shows that the presence of permeable boundary always leads to drag reduction compared to the channel with solid walls. When the permeability and the thickness of the fiber layer decrease, the drag reduction also decreases. In this case, for large values of α, there is a very small drag reduction in the channel. For α → ∞, the skin friction coefficient asymptotically approaches to the result of the pressure-driven flow in a channel with solid walls with no drag reduction. One interesting finding from Fig. 6(a,b) is that for each value of α, there is a range of δ values for which the maximum drag reductions in the channel occur.

### Theoretical validation and experimental methods

**Figure 7:**
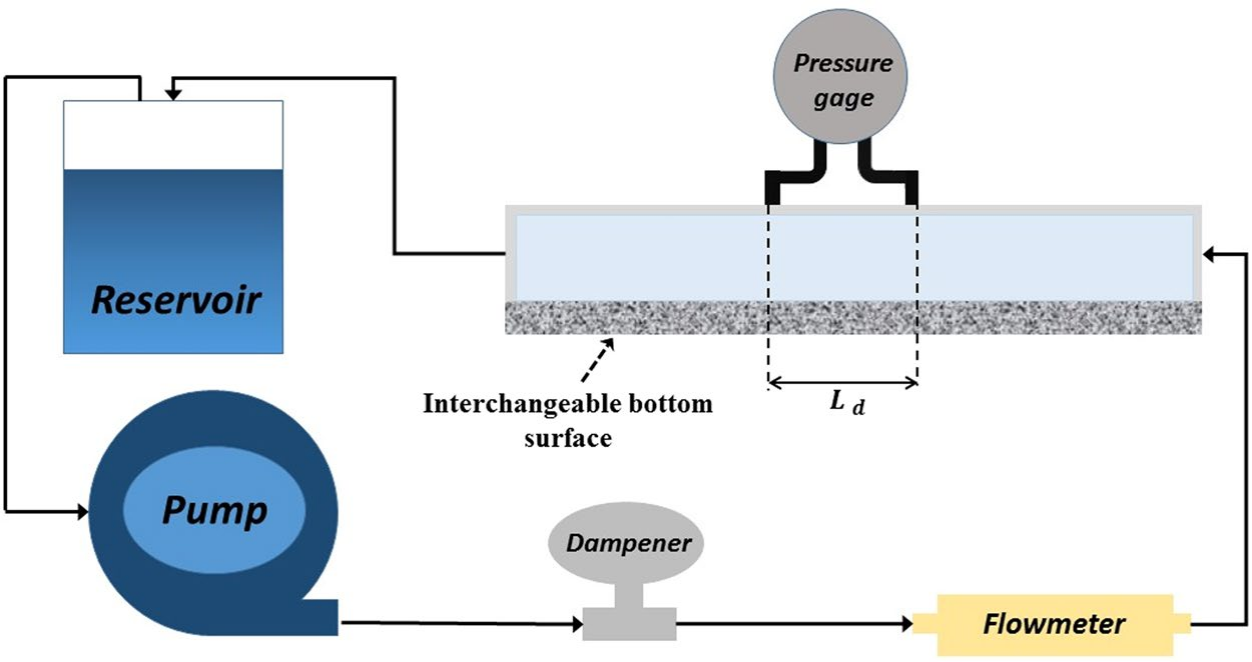
Schematic of the experimental setup for pressure drop measurements in the channel flow setup.

In order to validate our analytical predictions presented in previous sections, a series of experiments was conducted and the pressure drops across the channel for a range of conditions were measured. The schematic of the experimental set-up for pressure drop measurements is shown in Fig. 7. The experimental set-up was composed of a rectangular channel made of optically clear acrylic for flow visualization. The channel had a fixed height of 25 mm and a width of 42 mm. The working fluid consisted of 80% glycerin in 20% water, which was well stirred to maintain homogeneity as well as transparency. The resulting fluid had a viscosity and density of μ = 0.07 Pa.s and ρ = 1.21 g/cm^3^, respectively. A peristaltic pump (Simply Pump Inc.) was used to pump the fluid through the system. The pump had a maximum head of 20 ft. A flowmeter (Hedland Inc.; Range: 0.1~1.0 GPM) was also utilized for measuring the flow rate through the system. A dampener (BLACOH Fluid Control, Inc.) damped the effect of pulsatility of the pump during the experiments. A pressure gauge (Omega Inc.) with testing range 0~4.0 inH_2_O was used to measure the pressure drop in the channel. Interchangeable surfaces were placed on the bottom of the channel to adjust the height of the free fluid and to keep it constant during the test. In these experiments, the lower solid wall was shifted to accommodate for the permeable layer boundary conditions, while maintaining the same free flow channel height. The porous media used was manufactured by Mountain Mist that were made of random array of fibers with 5% silk and 95% polyester.

In order to obtain additional experimental data for various values of ${\rm{\delta }}=\frac{{\rm{H}}}{{\rm{L}}}$ and α, two different porous materials with the thickness of H = 4.2 mm and H = 5.5 mm, were used. Therefore, by controlling the height of free fluid section, different values of permeability parameter α and height ratio δ were obtained. We also measured the required entrance length for reaching fully developed velocity profiles in the channel to ensure that the flow enters the measurement zone under fully developed condition. Also, we maintained an average 5:1 cross-sectional aspect ratio for the channel to decrease the wall effects along the channel width. To keep the experimental condition the same as that of our analytical predictions, we have used two porous media with relatively low permeability K and high *α*. This was because as can be observed in Fig. 3(a–d) for large values of α (α > 10), the velocity profile has the characteristic of plug flow inside the porous media. Also, for large α values the fluid shear stress decreases to zero in the porous layer. Thus, we expect to perform the same results as in our theoretical analysis.

Before conducting the channel flow tests, we measured the Darcy permeability K, of the sample porous materials by performing the Darcy experiments. According to Darcy’s law

17 $$K=\frac{{\tilde{{\rm{Q}}}{\rm{L}}}_{{\rm{d}}}{\rm{\mu }}}{{\rm{A}}{\rm{\Delta }}\tilde{{\rm{P}}}},$$

where $\tilde{{\rm{Q}}}$ is the volume flow rate, L_d_ is the sample length over which the pressure drop occurs, and A is the cross-sectional area of the porous layer. The experimental measurements of Darcy permeability for the two porous media used in the experiments led to the values of 7.2 × 10^−8^ m^2^ and 6.2 × 10^−8^ m^2^. The corresponding porosities were measured to be 0.97 and 0.98.

Six sets of pressure drop experiments with different values of $\delta =\frac{H}{L}$ and $\alpha =\frac{L}{\sqrt{MK}}$ were conducted. Each set of experiments included two different tests: 1) a channel with solid walls, and 2) a channel with a porous wall. For these tests, the cross-sectional area of free flow section and the volume flow rates through the channel were identical. We also evaluated the Reynolds number for these tests based on the flow in the nonporous region. In these experiments, we measured drag reduction as^46^

18 $$DR{ \% }_{exp}\approx (1-\frac{{\rm{\Delta }}\tilde{{\rm{P}}}}{{{\rm{\Delta }}\tilde{{\rm{P}}}}_{s}}) \% \,$$

where ${{\rm{\Delta }}\tilde{{\rm{P}}}}_{s}$ corresponds to the measured pressure drop in channel with solid walls and ${\rm{\Delta }}\tilde{{\rm{P}}}$ is the measured pressure drop in the channel with one porous-wall. The predicted value of drag reduction, *DR*%_*theo*_, was evaluated by using the experimental permeability parameter *α* and height ratio *δ* in equations (3–10). The values of the height ratio *δ*, permeability parameter *α*, experimental drag reduction *DR*%_*exp*_, and predicted drag reduction *DR*%_*theo*_ are listed in Table 1. This table shows that the predicted drag reductions are in good agreement with those obtained from the experiments.

**Table 1:** The experimental parameters as well as predicted, and measured drag reduction.

Experiment #	1	2	3	4	5	6
*δ*	0.93	0.76	0.65	1.4	0.87	1.5
*α*	17	20	24	11	26	15
*DR*%_*theo*_	11.1%	9.5%	8.0%	16.6%	7.4%	12.5%
*DR*%_*exp*_	11.8%	10.4%	8.8%	15.0%	7.1%	12.5%

**Figure 8:**
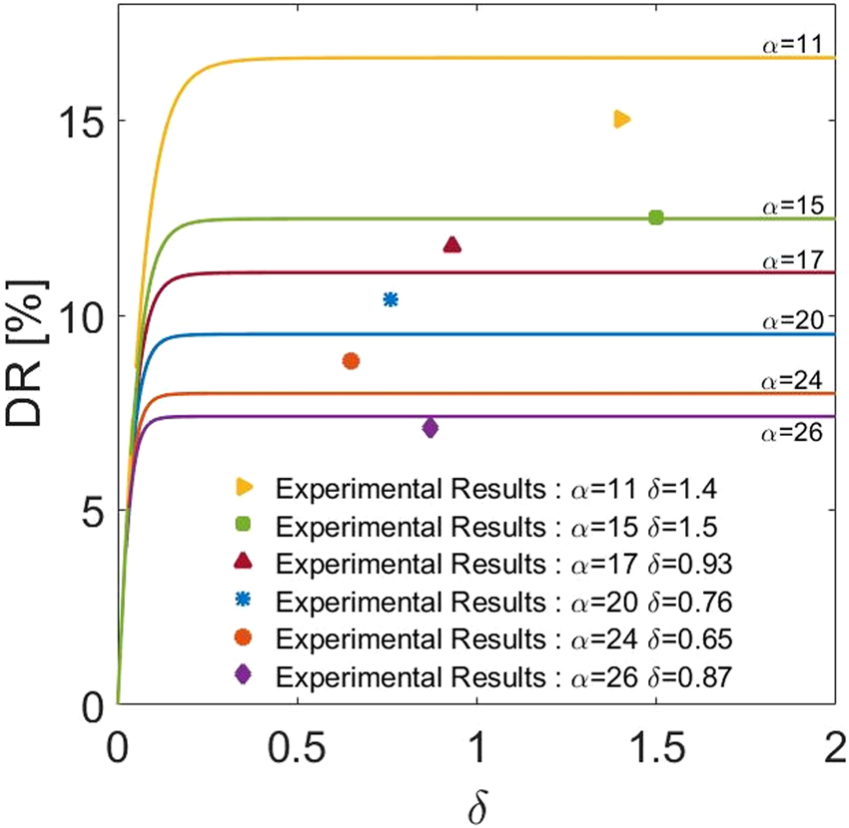
Comparison of the predicted drag reduction (solid lines) with the experimental data for the parameters listed in Table 1. The symbols represent experimental drag reductions.

Figure 8 also shows the comparison of the theoretical DR%_theo_ with the experimental DR%_exp_. The theoretical model predictions cover a range of values for *δ* and *α*. The experimental data were for specific values of parameter that are listed in Table 1 and are also shown in the figure by symbols. It is seen that the experimental data agree well with the theoretical predictions; however, there is some discrepancies particularly for smaller values of *α*. The deviations are attributed to the limitations in our study: 1) In the theoretical model it was assumed that the porous medium is distributed uniformly and the porous-fluid interface was smooth and at a fixed level. In the experiments, however, the distribution of fibers might have introduced variations in the level of fluid-porous media interface and also inhomogeneity of porosity or permeability near the interface, in addition to some surface roughness, 2) The experimental setup had a cross-sectional aspect ratio around (5:1), but in the analytical model the channel was considered to be two-dimensional and the 3D effects were neglected, 3) Correlations were made with Reynolds number that was based on the flow in the non-porous section. While this maybe a reasonable assumption for low permeability porous layer, it will introduce errors when permeability is high, and 4) The accuracy of the experimental measurement devices such as the pressure gauge and flow meter would also impact the experimental data.

## Conclusions

Laminar flows over permeable surfaces occur in various engineering applications^1,6,8–11^. Drag reduction in the presence of a porous boundary could significantly advance microfluidic-based devices in a wide range of industrial applications, in particular in biomedical fields. In this study, we developed an analytical framework for predicting laminar fluid flows and the corresponding drag reductions in ducts when the bottom solid surface of the channel is replaced with permeable layers. Particular attention was given to the case when the porosity of the porous layer is close to 1. Based on the presented results the following conclusions are drawn:

1. The presented results showed that the flow drag reduction was induced when the lower solid surface of a channel was replaced by a porous medium. The amount of drag reduction was shown to depend on the permeability parameter α = L/(MK)^1/2^ and the height ratio δ = H/L.
2. The limiting behavior of the flow over the porous boundary for both α → 0 and α → ∞ were evaluated. It was found that as α → ∞ the velocity and shear stress profiles asymptotically approaches to the limit of the pressure-driven flow in a channel with solid walls with height of 2 L. In the case when α → 0, the velocity and shear stress profiles becomes the same as those of a wider channel with a height of 2 L + H.
3. The model correlates the resulting reduction in drag to the characteristics of the permeable boundary surface. That is, with the knowledge of the channel geometry, the fluid bulk velocity, and the porosity and permeability of the porous wall, one can evaluate the drag reduction in the system.
4. The presented model reveals the relation between the physical properties of the permeable layer and the features of the fluid flows including drag reduction that can be used for designing relevant engineering systems. For example, knowing the values of the permeability parameter and the height ratio provides guidance for designing laboratory-scaled-model that are dynamically similar to their industrial scale prototypes.

In addition, for verifying the accuracy of the developed analytical model, several experiments were conducted for large values of α with specific height ratios and permeability parameters. Comparison of the results showed good agreements between the experimental data and analytical predictions for drag reduction.

Studying the effect of height variations of the fluid-porous media interface, and heterogeneity of porous media as well as the deformation of fibers and their interactions with the fluid flow are left for future studies.

### Data availability

The datasets generated during and/or analyzed during the current study are available from the corresponding author on reasonable request.
